# Competition between Free-Floating Plants Is Strongly Driven by Previously Experienced Phosphorus Concentrations in the Water Column

**DOI:** 10.1371/journal.pone.0162780

**Published:** 2016-09-13

**Authors:** Edwin T. H. M. Peeters, Rozemarijn E. M. Neefjes, Bastiaan G. van Zuidam

**Affiliations:** Aquatic Ecology and Water Quality Management Group, Wageningen University, Wageningen, The Netherlands; INRA, FRANCE

## Abstract

Nutrients can determine the outcome of the competition between different floating plant species. The response of floating plants to current phosphorus levels may be affected by previously experienced phosphorus concentrations because some species have the ability to store excess phosphorus for later use. This might have an impact on their competition. Here, we investigate the effect of previous and actual phosphorus concentrations on the growth rate of free-floating plant species (*Azolla filiculoides*, *Lemna minor/gibba* and *Ricciocarpus natans*)and the effect of phosphorus history on the competition between *L*. *minor/gibba* and *A*. *filiculoides* and between *L*. *minor/gibba* and *R*. *natans*. As expected, plant growth was lower when previously kept at low instead of high phosphorus concentrations. Growth of *L*. *minor/gibba* and *A*. *filiculoides* with a phosphorus rich history was comparable for low and high actual phosphorus concentrations, however, internal phosphorus concentrations were significantly lower with low actual phosphorus concentration. This indicates that both species perform luxury phosphorus uptake. Furthermore, internal P concentration in *Azolla* and *Lemna* increased within two weeks after a period of P deficit without a strong increase in growth. *A*. *filiculoides* in a mixture with *L*. *minor/gibba* grew faster than its monoculture. Morphological differences may explain why *A*. *filiculoides* outcompeted *L*. *minor/gibba* and these differences may be induced by phosphorus concentrations in the past. Growth of *L*. *minor/gibba* was only reduced by the presence of *A*. *filiculoides* with a high phosphorus history. Growth of *L*. *minor/gibba* and *R*. *natans* in mixtures was positively affected only when they had a high phosphorus history themselves and their competitor a low phosphorus history. These observations clearly indicate that phosphorus history of competing plants is important for understanding the outcome of the competition. Therefore, actual and previously experienced phosphorus concentrations should be taken into account in future studies dealing with competition between plants.

## Introduction

Free-floating macrophytes can have severe negative effects on aquatic ecosystems by forming dense floating mats. Several studies focused on the growth of free-floating plants in relation to phosphorus levels (e.g. [1, 2, 3, 4]). Among those studies, Kushari & Watanabe [1] found that species of the floating fern *Azolla* produced more biomass in P deficit conditions when the plants were previously held in enriched P conditions than when they were previously held in P deficit conditions. This indicates that these species could perform a luxury P uptake that can be used later for growth. If species take up additional phosphorus for later use, then the previous phosphorus concentration (or P history) in which plants have grown may be of major importance for their growth response to the actual phosphorus concentration. Free-floating aquatic plants are not rooted in the sediment and they are therefore completely dependent on the nutrient concentration in the water column [5]. Since free-floating plants compete with each other for among others nutrients [6, 7], the previous phosphorus concentration may affect the competition between these plants.

Small free-floating plants inhabit relatively small and shallow waters [8] like ponds, pools, small lakes, ditches and wetlands. Especially drainage ditches in agricultural areas often have high nutrient concentrations due to excessive loads of nutrients from the surrounding terrestrial environment, leading to frequent occurrences of free-floating plants like duckweed species [9]. The floating water fern *Azolla filiculoides* Lamarck (1783) is nowadays widespread in Europe [10] and competes with the common indigenous duckweed *Lemna minor/gibba* (L. 1753). As *L*. *minor/gibba* and *A*. *filiculoides* have similar growth forms, interference between these species is expected [11] and is probably influenced by nutrient availability in the water. *L*. *minor/gibba* occurs mostly in shallow meso- to eutrophic freshwaters, whereas *A*. *filiculoides* is often found in eutrophic environments [12]. The free-floating liverwort *Ricciocarpus natans* (L.) Corda, an indigenous red list species, is not common in the Netherlands and often occurs in the presence of *L*. *minor/gibba* in oligo- to mesotrophic conditions [12].

Global warming and eutrophication stimulate the growth of free-floating plants [9] resulting in dense mats that completely cover the surface of a water. As such mats are positioned at the water-atmosphere interface, they reduce the light penetration in the water column and the gaseous exchange, leading to lower dissolved oxygen concentrations under these mats [13]. This may then lead to lower aquatic biodiversity [14] due to fish kills, loss of invertebrates [13, 15] and loss of submerged macrophytes [13, 16, 17]. Replacement of mats of *L*. *minor/gibba* by mats of *A*. *filiculoides* is undesirable as the latter species appears to have even larger negative effects on the aquatic ecosystem than *Lemna* species [13].

*Azolla* species live in symbiosis with the nitrogen (N) fixating cyanobacteria *Anabaena azollae*, giving *Azolla* an advantage in N deficit conditions. *A*. *filiculoides* is therefore probably mostly limited by P availability [18, 19], whereas the growth of *L*. *minor/gibba* and *R*. *natans* is limited both by N and P. Several *Azolla* species have the capacity to perform luxury P uptake [1] and *A*. *filiculoides* might also have this capacity but this is still unclear. For *L*. *minor/gibba* and *R*. *natans* it is also unknown whether they can perform luxury P uptake and we are not aware of any reports of luxury uptake for these species or their genera. Excess uptake of P when it is available might lead to competitive advantages in later situations when the nutrient becomes limited.

Floating plants like *Azolla* and *Lemna* show a different response to temperature with *Azolla* growing faster than *Lemna* at lower temperatures and the reverse at intermediate temperatures [3]. Weather conditions vary from year to year being sometimes beneficial for one species and sometimes for the other. Furthermore, phosphorus concentrations in surface waters are variable over the year with, for example, a clear seasonal pattern for shallow lakes with low winter and high summer concentrations [20]. However, a pattern with high winter and low summer concentrations has also been observed and is depending on a number of other conditions [21]. These seasonal patterns in phosphorus also occur in drainage ditches [22] with the consequence that aquatic plants are faced with changing phosphorus concentrations over the growing season. Therefore, depending on the weather conditions, the composition of the competitors in the early phase of the growing season might be different and these competitors will face changing phosphorus conditions over time.

Overtopping is a strategy to outcompete other species and has been observed for floating plants [6]. For example, *L*. *minor* had the ability to overtop the submerged *Lemna trisulca* (L. 1753) and could win the competition [6]. In contrast to duckweeds, the fern *A*. *filiculoides* can grow not only horizontally but also vertically and this might give this species an advantage in the competition for light and space with *L*. *gibba/minor*.The objectives of this study are (1) to investigate the combined effects of the P history and the actual P concentration in determining the growth of *A*. *filiculoides*, *L*. *minor/gibba* and *R*. *natans*, (2) to determine the competition between *A*. *filiculoides* and *L*. *minor/gibba* and between *L*. *minor/gibba* and *R*. *natans* and (3) to investigate whether the P history influences this competition. To this end, the different species were cultivated under low and high P concentrations and thereafter grown under controlled laboratory conditions in either low or high P concentrations without and with their relevant competitors. To assess whether overtopping played a role in the competition, an experiment was performed to reveal the relationship between P concentration and morphology of *A*. *filiculoides*.

## Material and Methods

### General set up

**Fig 1:**
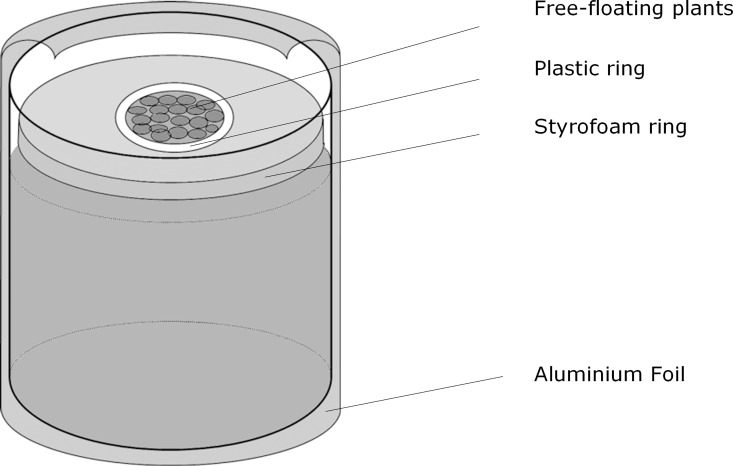
Schematic overview of experimental pots used in the experiment.

The effect of P history and present P was studied in an indoor laboratory experiment. In this experiment the growth of the 3 individual species as well as the competition between the species was studied. The experiment was carried out in a water bath in a climate controlled room. The water temperature of the water bath was 19.5 ± 0.5°C and the light intensity was on average 230 μmol m^-2^ s^-1^ PAR obtained from high-pressure sodium lights (Hortilux Schréder, HS2000, 400 Watt) with a 12h:12h day:night regime. Glass pots with a diameter of 7 cm were filled with 350 ml WC-medium [23] which was slightly adapted (4.2 instead of 14 mg N/L). A styrofoam ring with a circular hole (diameter 3 cm), lined with a plastic ring, floated on the water surface (Fig 1). In combination with aluminium foil on the outside of the pot this minimised the amount of light entering the water column and thus limited the growth of algae. Plants were placed on the remaining water surface. The pots were placed randomly in the water bath and the duration of the experiment was 2 weeks. Fresh weight of the plants was measured at the beginning and at the end of the experiment to determine the change in biomass. 50 ml Water samples were taken at the start and end of the experiment to determine N and P concentrations in the water. The samples were analysed photometrically with a Continuous Flow Analyser (Skalar Analytical BV, Breda, The Netherlands) following the standard protocols for P and N [24, 25, 26]. Enough nutrients were left at the end of the experiment to support plant growth (see S1 and S2 Tables). During the experiment the growth of the plants was monitored by daily observations. Total N and P were determined in monocultures of the plants at the end of the experiment from all four treatments in case enough material was available. Total N and P concentrations in plants were determined on a segmented flow analyser after destruction with sulphuric acid/salicylic acid/selenium/hydrogen peroxide, with total N measurement based on the Berthelot reaction and total P measured as phosphate molybdenum [27, 28].

### Plant species

*A*. *filiculoides* (further *Azolla*) was collected in Zetten (51° 55’ 29.0” N, 5° 43’ 21.1” E), *L*. *minor/gibba* (further *Lemna*) in Wageningen (51° 59’ 44.2” N, 5° 36’ 5.9” E) and *R*. *natans* (further *Ricciocarpus*) in Valburg (51° 55’ 27.9” N, 5° 45’ 57.1” E) in the Netherlands. No specific permission was required for collecting the plants since the sites were located in public areas. The species used in this study are not endangered or protected species. The collected species were cultivated in the water bath in the climate controlled room in 5 L WC-medium [23] that was slightly modified with 0 mg N/L for *Azolla* and 4.2 mg N/L for *Lemna* and *Ricciocarpus*. The plants were cultivated under the same conditions as those that were used in the experiment. The medium of the cultures was replaced weekly by pouring off the medium and retaining the plants on a sieve (0.5 mm). Plants were rinsed with a showerhead, mimicking a rain shower, to remove any algae or dead material. Prior to the experiment plants were in the laboratory for at least 6 months.

### P treatments

12 Weeks prior to the start of the experiment, the 3 species received a pre-treatment in which they were either cultivated at a high P concentration of 1.55 mg P/L or at a low P concentration of 0.03 mg P/L together with 4.2 mg N/L. Pre-experiments showed that the growth of the plants was not limited at the high P concentration, whereas at the low concentration their growth was limited without causing them to die in this 12 week period. Plants cultured in the low or the high P concentration in the pre-treatment are referred to as plants with a low or a high *P history*, respectively. During the main experiment two P treatments were applied, which are referred to as high and low *present* P (1.55 and 0.03 mg P/L respectively). The four possible combinations of the two P *histories* and the two *present* P were tested in the experiment for each monoculture separately and for the relevant combinations of competing species (*Azolla* with *Lemna* and *Lemna* with *Ricciocarpus*) which resulted in a total of 28 treatments (Table 1). At the start of the experiment, 100% of the water surface was covered by the species in the treatments with the monocultures, whereas in the competition treatments each species covered 50% of the surface. The fresh weight of 100% coverage of the water surface was determined for each species and *P history* prior to the experiment. The plants were randomly taken from the cultures. The mean (± stdev) weights of the plants to cover 100% surface with a low *P history* were 0.2361 ± 0.0200 g, 0.4227 ± 0.0392 g and 0.4406 ± 0.0130 g for *Lemna*, *Azolla* and *Ricciocarpus*, respectively, and with a high *P history* the weights were 0.2199 ± 0.0157 g, 0.4441 ± 0.0419 g and 0.3421 ± 0.0238 g, respectively. These weights were used at the start of the experiment for the monocultures and 50% of the weights was used for the competition treatments.

**Table 1:** Overview of treatments for monocultures *of A*. *filiculoides*, *L*. *minor/gibba* and *R*. *natans* and the relevant competing species (*A*. *filiculoides* & *L*. *minor/gibba* and *L*. *minor/gibba* & *R*. *natans*). Between brackets are the number of replicates.

Species	P history	*Azolla*		*Lemna*		*Ricciocarpus*	
		Present P		Present P		Present P	
		Low	High	Low	High	Low	High
*Azolla*	Low	A	A	B	B		
		(4)	(4)	(4)	(4)		
	High	A	A	B	B		
		(4)	(4)	(4)	(4)		
*Lemna*	Low	B	B	A	A	C	C
		(4)	(4)	(4)	(4)	(1)	(1)
	High	B	B	A	A	C	C
		(4)	(4)	(4)	(6)	(2)	(2)
*Ricciocarpus*	Low			C	C	A	A
				(2)	(2)	(2)	(2)
	High			C	C	A	A
				(2)	(2)	(2)	(2)

A: Treatments of monocultures.

B: Treatments of competition between *Azolla* & *Lemna*.

C: Treatments of competition between *Lemna* & *Ricciocarpes*.

All treatments were replicated four times, except those containing *Ricciocarpus* which were done in twofold. The treatments with monocultures of *Azolla* and *Lemna* and the treatments with the competition of these two species were carried out in two separate time blocks, with two replicates per block.

### Biomass measurements

Prior to weighing, plants were rinsed to remove algae and dead material and were put onto a tissue and blotted dry carefully. After drying for approximately 2 minutes they were weighed on an analytical scale to determine the fresh weight. The species in the mixed cultures were separated from each other by hand and each species was weighed separately.

### P treatment and morphology

An additional experiment was carried out to quantify the plant morphology of *Azolla*. *Azolla* with a low P *history* and *Azolla* with a high P *history* were subsequently grown in medium with a high *present* P. These P treatments were combined with two density treatments, as the experiment started either with 100% coverage or with 2 individuals in a pot. All other conditions during this experiment were identical to those in the main experiment. All combinations were carried out in fourfold, leading to 16 pots in total. The plants were grown for 2 weeks, after which the height of the mat (100% treatment) or the plants (2 individuals treatment) were measured. In the treatments with 2 individuals, the height of the plants was measured as the distance between the water surface and the highest point of the plant. In the treatment with 100% coverage, the height of the mat was measured the same way, but was determined by averaging the height of 9 random points of the mat. Furthermore, in all treatments the length and width of the plants were measured per individual plant. To compare the height of *Azolla* with that of *Lemna*, the height of 20 healthy *Lemna* plants with a high *P history* and high *present P* was measured as well.

### Data analysis

The relative growth rate (RGR) of the species was calculated as:

$$RGR=\frac{LN({FW}_{2})-LN({FW}_{1})}{{(t}_{2}-t_{1})}$$ (1)

in which FW_1_ and FW_2_ are fresh weights (expressed as mg) measured at start t_1_ and end t_2_ of the experiment (in days), respectively, and can be found in S3 Table.

Statistical analysis was performed using IBM SPSS statistics 22. A preliminary analysis of the data showed that the linear model and ANOVA could be used, as the untransformed data did not deviate from the normal distribution and the variance was distributed homogeneously over the treatments. A linear mixed model showed that the random effect of the two time blocks did not significantly influence the fit of the model (z = 0.267, p = 0.789) and, therefore, conventional ANOVAs based on the linear model were used for the statistical analysis. Three different types of analyses were carried out, focussing on three different aspects of the results: differences in maximum growth between species, the effect of the P treatments on each monoculture and the combined effect of competition and the P treatments. Firstly a single ANOVA was used to test whether the maximum growth rate differed between the three species. For this, the RGR in the treatment with high *P history* and high *present P* was compared between the species. Secondly, the effect of the P treatments on the monocultures was analysed for each species separately using ANOVA. In this analysis, the RGR of a monoculture was used as a response and *P history*, *present P*, and their interaction were used as explanatory factors. Pairwise comparisons were made between all treatments within each species. Thirdly, the combined effect of competitors and the P treatments was analysed per species using ANOVA. In this analysis, the RGRs of one species in the monocultures and in the competition treatments were used as the response and the *P history*, the *present P*, the competitor, the *P history* of the competitor and the interactions between these factors were used as explanatory factors. For *Lemna* and *Azolla*, the effect of the presence of a competitor and the *P history* of this competitor was determined by making pairwise comparisons between the growth rate in the treatments with and without the competitor and with different P histories of the competitor. These pairwise comparisons were made within each of the four combinations of *P history* and *present P* of the species for which the RGR was measured. The same approach was used for *Ricciocarpus*, but for this species pairwise comparisons were made only within each of the two P histories of *Ricciocarpus*, not differentiating between the present P treatments. Oneway ANOVA per species was used to test whether the treatments resulted in significant differences in N or P concentrations within plants.

## Results

### P treatment and species’ growth

**Fig 2:**
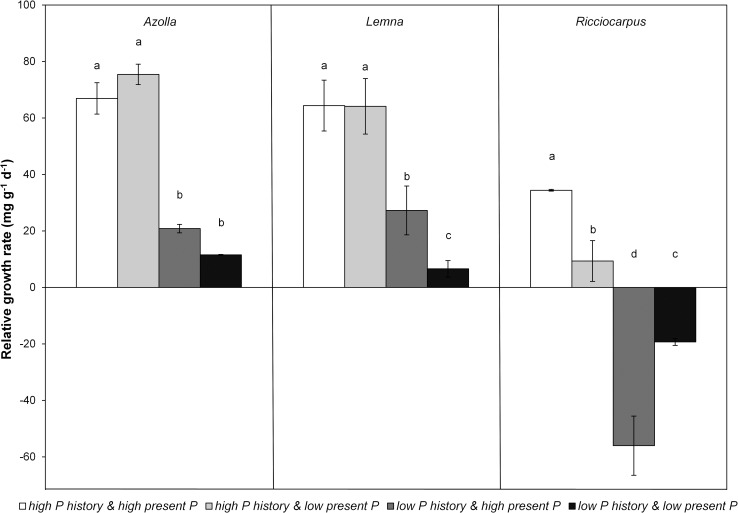
Mean (± standard deviation) relative growth rate (RGR) of monocultures of *Azolla (A*. *filiculoides)*, *Lemna (L*. *minor/gibba*, and *Ricciocarpus (R*. *natans)* in the four phosphorus treatments. Different letters indicate significant differences (p<0.05) found through pairwise comparisons. Pairwise comparisons were done within each species.

There were significant differences between the maximum growth rates of the three species grown at a *high P history* and a *high present P* (F_2,11_ = 19.706, p<0.001; Fig 2). Growth rate of *Ricciocarpus* was approximately half that of *Lemna* and *Azolla* and the latter two did not differ from each other. The combination of P history and present P determined the growth response of the monocultures (F_9,30_ = 78.339, p<0.001). There was a large difference between the RGRs of the monocultures and this difference was related to P history: a *low P history* resulted in slower growth than a *high P history* (Fig 2). *Lemna* and *Azolla* had a similar growth response to the present P treatments with no observed differences in growth rates between a *high present P* and a *low present P* when they had a *high P history*. Thus, the present P did not influence the RGR of these species when they had a *high P history*. Whereas for *Azolla* the present P did not influence the RGR after a *low P history* either, the growth of *Lemna* with a *low P history* was affected by present P. The latter species had a significantly smaller RGR in *low present P* than in *high present P* when pre-treated with *a low P history* (pairwise comparisons p = 0.009). The growth rates of *Ricciocarpus* showed a different pattern compared to the other two species. *Ricciocarpus* with a *high P history* grew significantly slower in *low present P* compared to *high present P* (pairwise comparions p<0.001), showing that the RGR of this species was immediately affected by the present P. Furthermore, this species was affected more strongly by the P history compared to the other two species, as the RGR of *Ricciocarpus* was negative when it had a *low P history*, both in *low* and *high present P*.

**Fig 3:**
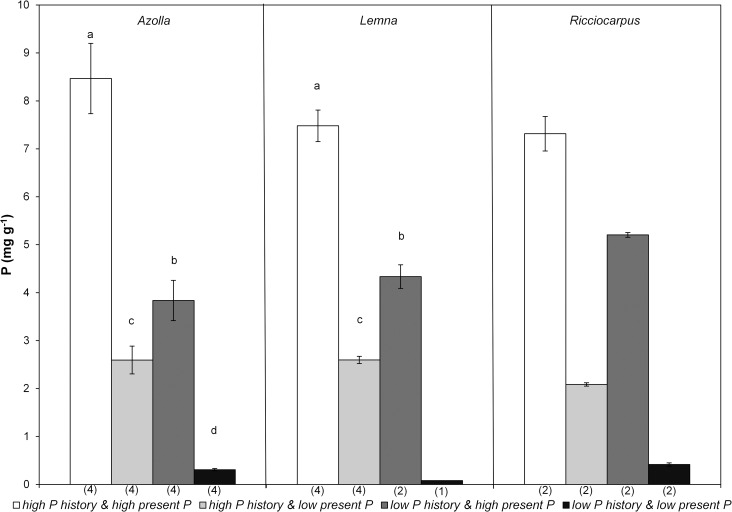
Mean (± standard deviation) phosphorus concentration in the plants of the monocultures of *Azolla* (*A*. *filiculoides*), *Lemna* (*L*. *minor/gibba*) and *Ricciocarpus* (*R*. *natans*) in the four treatments at the end of the experiment. Different letters indicate significant differences (p<0.05) found through pairwise comparisons. Number of replicates is given between brackets below the bars.

**Fig 4:**
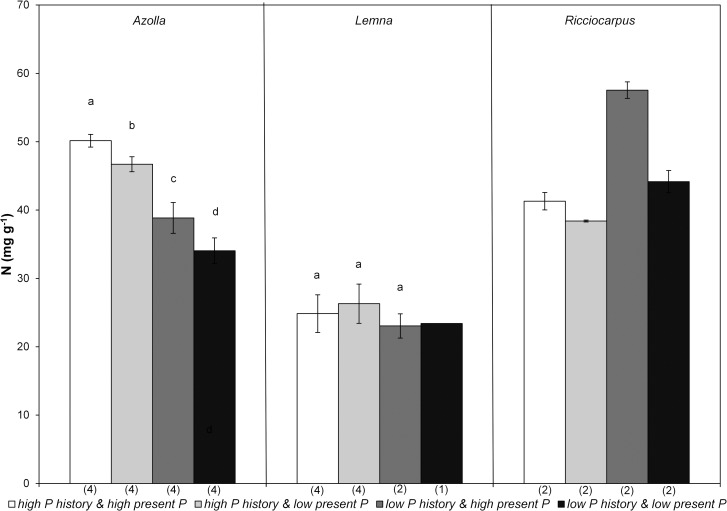
Mean (± standard deviation) nitrogen concentration in the plants of the monocultures of *Azolla* (*A*. *filiculoides*), *Lemna* (*L*. *minor/gibba*) and *Ricciocarpus* (*R*. *natans*) in the four treatments at the end of the experiment. Different letters indicate significant differences (p<0.05) found through pairwise comparisons. Number of replicates is given between brackets below the bars.

The amount of plant material was not always sufficient to analyse N and P in the plants resulting in low number of replicates for some treatments (see S4 Table). As expected, highest P concentrations in plants were observed in the treatment *high P history* and *high present P* while lowest values were observed in *low P history* and *low present P* (Fig 3). All four tested treatments were significantly different for *Azolla* (F_3,15_ = 961.528, p<0.000) and all three for *Lemna* (F_2,9_ = 766.802, p<0.000), however, the number of replication was too low for statistical analysis for *Ricciocarpus*. All three species showed a higher internal P concentration in the treatment *low P history* and *high present P* than in the treatment *high P history* and *low present P* and this internal concentration was much higher than in the treatment *low P history* and *low present P*. N content (Fig 4) in *Azolla* was also significantly different between the four treatments (F_3,15_ = 80.612, p<0.000) but this was not the case for *Lemna* (F_2,9_ = 0.992, p = 0.418).

### P treatment and competition

**Fig 5:**
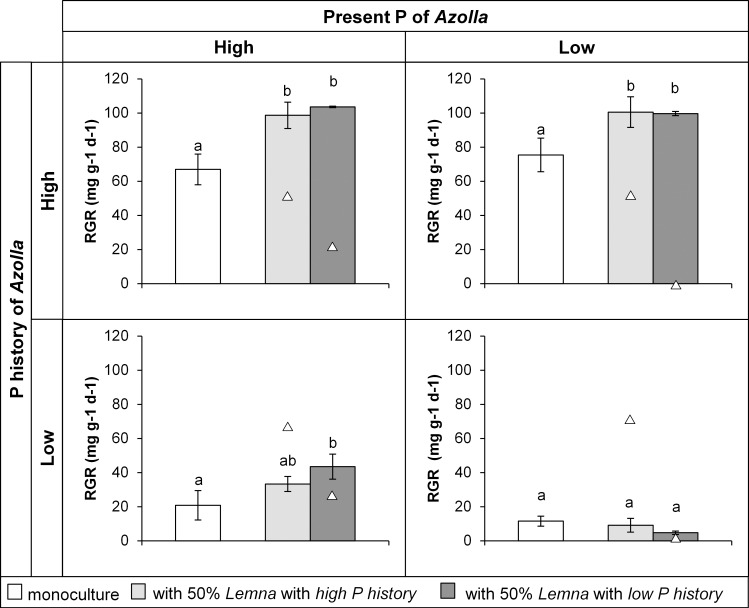
Mean (± standard deviation) relative growth rate (RGR) of *Azolla* (bars) in the four phosphorus treatments when grown as monoculture and in competition with *Lemna* (triangles represent mean RGR of the competitor). In the competition treatments, 50% of the surface was initially covered by *Azolla* (*A*. *filiculoides)* and 50% by *Lemna* (*L*. *minor/gibba)*. Different letters within each panel indicate significant differences (p<0.05) found through pairwise comparisons.

The effect of the competitor on the growth of *Azolla* depended on the treatment of *Azolla* itself (F_6,28_ = 6.833, p<0.001; Fig 5). The presence of *Lemna* did not affect the growth of *Azolla* in case *Azolla* grew in *low present P* and had a low *P history* (Fig 5). However, the RGR of *Azolla* in *high present P*, was positively influenced by the presence of *Lemna* with a *low P history* (Fig 5, pairwise comparisons p = 0.002). This was also the case for *Azolla* with a *high P history* (Fig 5), which showed a larger growth in combination with *Lemna* in both present P treatments, regardless of the P history of *Lemna*. Therefore, the intraspecific competition for *Azolla* is more severe than the interspecific competition with *Lemna* plants. Interestingly, the P history of *Lemna* had no significant effect on the RGR of *Azolla*.

**Fig 6:**
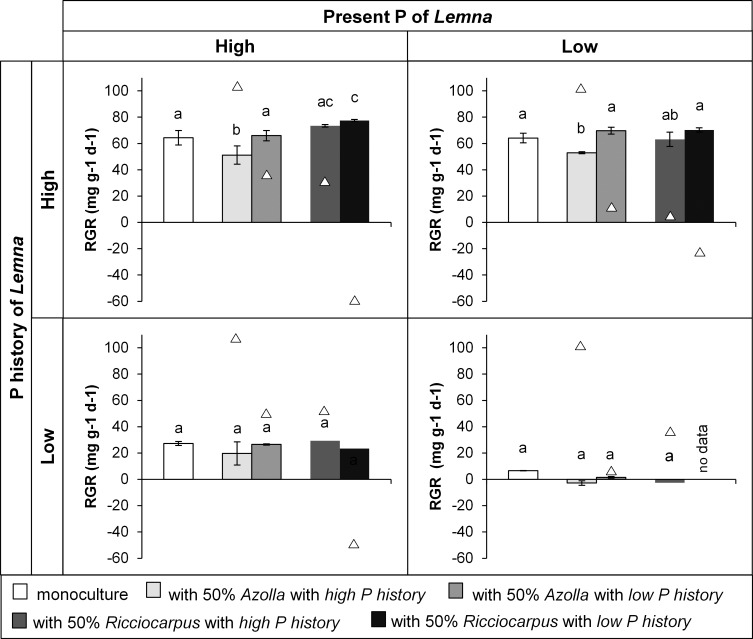
Mean (± standard deviation) relative growth rate (RGR) of *Lemna* (bars) in the four phosphorus treatments when grown as monoculture and in competition with *Azolla* or *Ricciocarpus* (triangles represent mean RGR of the competitor) In the competition treatments, 50% of the surface was initially covered by *Lemna (L*. *minor/gibba*) and 50% by either *Azolla* (*A*. *filiculoides*) or *Ricciocarpus* (*R*. *natans*). Different letters within each panel indicate significant differences (p<0.05) found through pairwise comparisons.

The growth of *Lemna* was affected by the presence of a competitor and by the P history of this competitor (F_11,34_ = 2.807, p = 0.010), but the effect depended also on the treatment of *Lemna* itself as was the case for *Azolla*. The RGR of *Lemna* with a *low P history* was not affected by the presence of a competitor, whereas an effect was observed when *Lemna* had a *high P history* (Fig 6). *Lemna* with a *high P history* grew significantly slower in the presence of *Azolla* with a *high P history* compared to its corresponding monoculture both for *low present P* (pairwise comparisons p<0.001) and *high present P* (pairwise comparisons p = 0.003). Thus, the two treatments with *Azolla* from a *high P history* had a negative effect on the RGR of *Lemna*, while there was no effect of *Azolla* from a *low P history*. Such a pattern seemed also present for *Lemna* with a *low P history* but the differences were not significant. *Lemna* with a *high P history* responded differently to the presence of *Ricciocarpus* than to the presence of *Azolla*. *Lemna* grew faster in *high present P* in combination with *Ricciocarpus* with a *low P history* compared to the monoculture ((Fig 6) pairwise comparisons p = 0.004), indicating that the competition with *Ricciocarpus* is weaker than the competition with specimens of its own kind. Although this pattern also seemed to be present for *Lemna* in combination with *Ricciocarpus* with a *high P history* and in *low present P* for *Lemna* with a *high P history* in combination with *Ricciocarpus* with a *low P history*, these differences were not significant.

**Fig 7:**
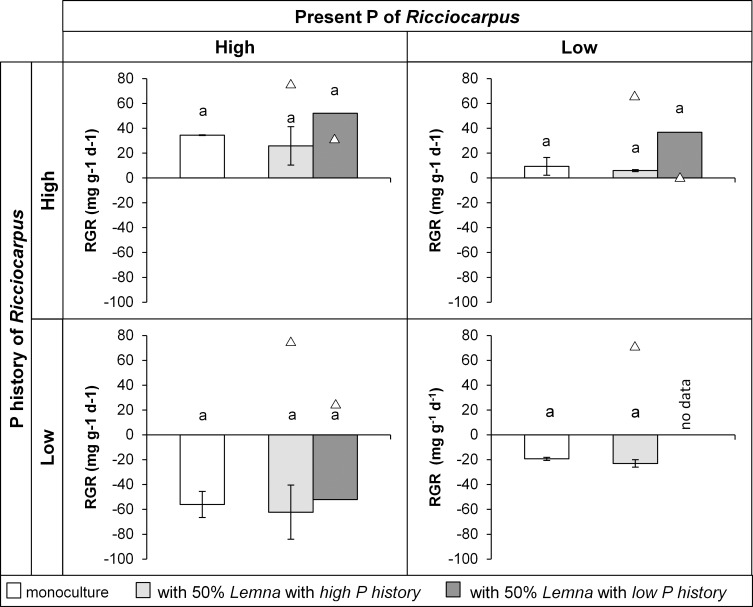
Mean (±standard deviation) relative growth rate (RGR) of *Ricciocarpus* (bars) in the four phosphorus treatments when grown as monoculture and in competition with *Lemna* (triangles represent mean RGR of the competitor). In the competition treatments, 50% of the surface was initially covered by *Ricciocarpus* (*R*. *natans*) and 50% by *Lemna* (*L*. *minor/gibba*). Different letters within each panel indicate significant differences (p<0.05) found through pairwise comparisons.

Despite the limited number of replications for treatments with *Ricciocarpus* it is obvious that the response of this species to the presence of *Lemna* did not depend on the treatment of *Ricciocarpus* itself (Fig 7). It reacted the same way to *Lemna* in all four treatments. Its growth, however, did depend on the presence of *Lemna* and on the P history of this *Lemna* (F_2,13_ = 6.010, p = 0.014). There is a significant difference between the grow of *Ricciocarpus* in combination with *Lemna* with a *high* and with a *low P history* (pairwise comparisons p = 0.012). *Ricciocarpus* grown in the presence of *Lemna* with a *low P history* had a larger growth than *Ricciocarpus* in the monoculture (pairwise comparisons p = 0.063). On the other hand, growth of *Ricciocarpus* did not differ from the monoculture when it grew in combination with *Lemna* with a *high P history*.

### P treatment and morphology

**Fig 8:**
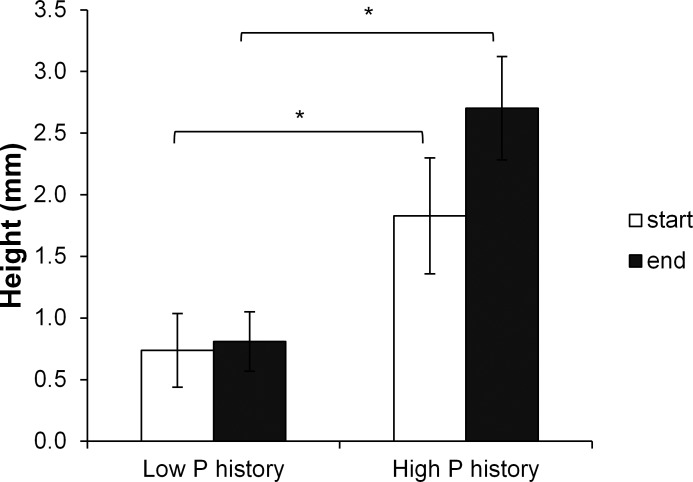
Height of *A*. *filiculoides* plants with a low previous phosphorous concentration (*low P history*) and with a high previous phosphorous concentration (*high P history*) at the start of the experiment and after 2 weeks of growth in medium with a high phosphorous concentration. The brackets indicate the heights that have been compared and the stars indicate significant differences (p<0.05) determined by ANOVA.

After the pre-treatment there was a clear difference in the mean (± stdev) height between plants with a *high P history* (2.2 ± 0.2 mm) and those with a *low P history* (0.6 ± 0.4 mm) and this difference was significant (F_1,14_ = 30.743, p<0.001; Fig 8). Interestingly, after two weeks of growth in water with a *high present P*, this height difference was still there (F_1,14_ = 122.825, p<0.001; Fig 8). The plants with a *low P history* had not grown much taller, whereas the height of those with a *high P history* had increased, leading to an even larger height difference between the plants with the different P histories. This was the case for the mat as well as the individual plants. Furthermore, at the end of the experiment both width and length of the *Azolla* plants were significantly smaller (4.6 ± 2.3 versus 11.5 ± 3.2 mm for width and 7.3 ± 2.2 versus 12.7 ± 5.0 mm for length; F_1,164_ = 91.474, p<0.001; F_1,164_ = 219.984, p<0.001, respectively) when they had a *low P history* compared to those with a *high P history*. This shows that the morphology of *Azolla* is strongly influenced by its P history and that this effect of P history persists far longer than two weeks. Healthy *Lemna* (average length 4.8 ± 0.6 mm) was not higher than 0.4 ± 0.1 mm (n = 20) and can easily be overtopped by *Azolla* held under P rich conditions.

## Discussion

### P treatment and species’ growth

The growth rates of monocultures of *L*. *minor/gibba* and *A*. *filiculoides* were similar to each other when grown in conditions were light and nutrients were not limiting, while *R*. *natans* grew approximately twice as slow. The RGRs of all three species were influenced by both the phosphorus concentration to which the plant was exposed in the past and the actual phosphorus concentration. Interestingly, it was the phosphorus history that largely determined the growth response of the free-floating plants to the present concentration, demonstrated by plants with a phosphorus rich history having a much higher growth rate. This supports the results of Kushari & Watanabe [1] who observed that plants of other *Azolla* species first grown in a phosphorus enriched medium obtained a larger biomass when held in a phosphorus deficit medium than plants that were first starved of phosphorus. This is supported by results from the present study where the growth of *L*. *minor/gibba* and *A*. *filiculoides* was not influenced by the actual (low or high) phosphorus concentration when they were previously grown in a high phosphorus concentration. Interestingly, the internal P concentrations were much lower when the species were exposed to low actual phosphorus concentrations, and these internal concentrations were still much higher than those from a previous and actual low phosphorus concentration. In addition, their growth was much less in low than in high actual phosphorus concentration when they were held in low phosphorus in the past. A possible explanation for these observations might be that both *A*. *filiculoides* and *L*. *minor/gibba* store additional phosphorus in their tissue when grown in phosphorus rich water. They likely use this stored phosphorus when the actual available concentration is low to maintain a growth that is similar to the growth in a phosphorus rich environment. This luxury phosphorus uptake is a mechanism known to occur in other aquatic plants [29] and in other *Azolla* species [30, 31]. Since the growth rate of *R*. *natans* was immediately negatively affected by a low actual phosphorus concentration, even when the liverwort was previously held on phosphorus rich medium, luxury phosphorus uptake seems not likely in this species. Our observations thus confirm the view that phosphorus storage capacity is species specific [29] and that, as a result, the combined effect of the phosphorus history and the actual phosphorus concentration differs per species.

Our results show that species may recover from exposure to low phosphorus concentrations. For example, *L*. *minor/gibba* previously held under low phosphorus conditions and grown in a high actual phosphorus concentration, shows signs of recovery since visual observations during the experiments demonstrated that the plants turned greener and the fronds became a bit larger but not visibly higher. The larger growth rate as compared to the same treatment with a low actual phosphorus concentration also indicated recovery. Furthermore, even after two weeks the RGR was nowhere near the RGR of the species that were held in phosphorus rich conditions in the past. The recovery was thus not immediate and apparently the plants need much more time to adapt to the higher P concentration. Interestingly, the internal phosphorus concentration increased during these two weeks without a strong increase in growth. A similar pattern was observed in the present study for *A*. *filiculoides*, but not for *R*. *natans*. Phosphorus starved *R*. *natans* did not recover at all and in fact transferring these plants to a higher actual phosphorus concentration resulted in a further decrease of biomass. Keeping *R*. *natans* for a longer period on phosphorus poor water resulted in a too low quality of *R*. *natans* to recover from this stressful environment. The low productivity of *R*. *natans* under these conditions went along with observed algal growth in this treatment. The plants floated just below the water surface, leading to a thin layer of water above the plants in which the algae were able to grow, thereby making use of the available P. The presence of algae partly covering a free-floating plant can reduce the growth of this plant [32]. This overgrowth by algae was only observed in the monoculture *R*. *natans* and did not occur in the treatments of *L*. *minor/gibba* or *A*. *filiculoides*.

### P treatment, competition and morphology

Our results indicate that in mesotrophic to eutrophic conditions A. *filiculoides* may outcompete *L*. *minor/gibba*, and *L*. *minor/gibba* may outcompete *R*. *natans*. Phosphorus conditions in the past play a crucial role in the competition between these free-floating plants. For the competition between *L*. *minor/gibba* and *A*. *filiculoides* we observed that the effect of the competitor depended on their own phosphorus history. For example, when *L*. *minor/gibba* was cultured under low phosphorus conditions the competitors did not influence the growth rate, whereas they did when *L*. *minor/gibba* was previously held in a high phosphorus concentration. Thus, the competition became more pronounced in our study when there was a shift from a nutrient limited situation to a space or light limited situation. This supports the resource competition theory, according to which competitive exclusion of less competitive species mainly occurs when resources other than space and light are no longer limiting [33, 34]. In contrast with this, is the competition between *L*. *minor*/gibba and *R*. *natans*, where the extent of the impact of the competitor nearly completely depends on the phosphorus history of the competitor.

Surprisingly, the RGR of *A*. *filiculoides* was higher when it grew in the presence of *L*. *minor/gibba* compared to the monoculture, and this was regardless of the phosphorus history of *L*. *minor/gibba*. This means that the growth of *A*. *filiculoides* is reduced more by the presence of plants of its own kind than by that of *L*. *minor/gibba*. A possible reason for this could be the morphology of the plants [6, 7, 35, 36]. The leaves of *L*. *minor/gibba* are small, lay flat on the water surface and expand mostly in horizontal direction, whereas the leaves of *A*. *filiculoides* have multiple branches and expand in both horizontal and vertical direction. In monocultures, *A*. *filiculoides* limits its own growth when crowded. In the presence of *L*. *minor/gibba A*. *filiculoides* has more space to grow, as it can grow over the leaves of *L*. *minor/gibba* and grow upward to occupy more space. Visual observations show that the diameter of the fronts of *L*. *minor/gibba* may change a little bit under influence of different phosphorus histories, whereas the height did not. This could explain why the growth of *A*. *filiculoides* is not affected by the phosphorus history of *L*. *minor/gibba*. Unlike *L*. *minor/gibba*, the morphology of *A*. *filiculoides* is strongly influenced by its own phosphorus history. According to the results of our morphology experiment, *A*. *filiculoides* plants were larger and higher when they were held in a high phosphorus concentration in the past than in a low phosphorus concentration in the past. This could well explain why *A*. *filiculoides* with a high phosphorus history had a strong, negative influence on *L*. *minor/gibba*, whereas *A*. *filiculoides* with a low phosphorus history hardly influenced the growth of *L*. *minor/gibba*. *A*. *filiculoides* with a low P history had small leaves that hardly affected the growth of *L*. *minor/gibba* but *A*. *filiculoides* with a high P history strongly declined the growth of *L*. *minor/gibba* indicating that *A*. *filiculoides* is the better competitor for light. A similar result was found by Dickinson & Miller [7], who attributed the success of *Salvinia minima* (Baker 1996) to outcompete *Spirodela punctata* (L.) Schleid. (1839) and *Azolla caroliniana* Kaulf (1824) to its size and height, by overtopping the other two species. It is likely that *A*. *filiculoides* with a high phosphorus history will be able to outcompete *L*. *minor/gibba* after a longer time period than these two weeks, as it might completely overgrow *L*. *minor/gibba*. Overall, the phosphorus concentration to which plants were exposed in the past, hugely influences the morphology of the plants, which is a likely explanation for the different responses of the free-floating plants during competition.

The competition between *R*. *natans* and *L*. *minor/gibba* is different from that between *L*. *minor/gibba* and *A*. *filiculoides*. *R*. *natans* was not affected differently by the presence of *L*. *minor/gibba* with a high phosphorus history than by its own fronds, which could be explained by the similar morphology of the two species. Both species lie flat on the water surface and mostly grow horizontally. These similarities can be the reason for their frequently observed coexistence in mesotrophic waters [12] since weak competitors and neutrality do not lead to competitive exclusion. Furthermore, the observations showed that the species with a high phosphorus history (either being *L*. *minor/gibba* or *R*. *natans*) usually outcompeted the other species with a low phosphorus history, a phenomenon that was also observed by others [37]. In the present study this difference in competitiveness could be related to the condition of the plants.

### Implications

Various studies investigated the relationship between free-floating plants and phosphorus levels experimentally (e.g. [3, 4, 19, 38]). This current study shows that the phosphorus concentration in the past largely determines the response of small, free-floating plant species to the actual phosphorus concentration. The phosphorus history thus strongly influences the outcome of exposure to current phosphorus concentrations, but to our knowledge studies focussing on floating plants and phosphorus did not take this into account. Therefore, in future studies it is very important to be aware and to take this phosphorus history into account to avoid bias. This is especially the case when species, like *L*. *minor/gibba* and *A*. *filiculoides*, may perform luxury phosphorus uptake as this can strongly influence the outcome of a study.

This research shows that it takes quite some time for a species to adapt to the current phosphorus concentration, both when it is lower or higher than the phosphorus concentration in the past. This may have consequences for management to control free-floating plant biomass. Flushing with nutrient poor and oxygen rich water is a measure to reduce the negative effects of thick floating plant mats. Although the oxygen might be beneficial for fish stock and other animal life, the low nutrient levels will not have a strong, immediate effect on *L*. *minor/gibba* or *A*. *filiculoides* due to their luxury P uptake. The plants will be able to keep on growing, despite the lower nutrient concentrations.

Climate conditions in the winter are largely determining the onset of mats of free-floating plants in temperate regions [9, 39], however, the phosphorus concentrations at the start of the growing season are important as well. Available P concentrations are frequently higher in winter and at the start of the growing season than in summer. To understand the occurrence of problematic floating mats in summer, it may be necessary to take into account the nutrient concentrations that occurred earlier in the year. As shown in this research, the phosphorus concentration may influence the morphology of the plant, thereby influencing the competition ability of those plants in the future. Plants that are able to make optimal use of available phosphorus at the beginning of the growing season and even store phosphorus, have a competitive advantage over plants that are not able to do so.

## Conclusion

This study shows that the phosphorus concentration to which free-floating plants have been exposed in the past (the P history) largely determines how the growth of a plant reacts to the actual phosphorus concentration. Therefore, it is important to take the phosphorus history into account in future studies. There are clear indications that *A*. *filiculoides* and *L*. *minor/gibba* may perform luxury phosphorus uptake, whereas this is clearly absent in *R*. *natans*. This additional phosphorus may support high growth rate when plants become exposed to low phosphorus concentration and as a result the internal concentration drops. This study also clearly indicates that the internal phosphorus concentration in both *Azolla* and *Lemna* increased during the two weeks of experimenting without a strong increase in growth. Morphological characteristics play an essential role in the competition between the species used in this study. The phosphorus history determines the morphology and thereby influences the competition. Under previous phosphorus rich conditions *A*. *filiculoides* is a stronger competitor compared to *L*. *minor/gibba*, because its morphology makes it a better competitor for light and space. *L*. *minor/gibba* grows better in all phosphorus treatments than *R*. *natans*. *L*. *minor/gibba* and *R*. *natans* have similar morphological characteristics which are less affected by the P history. Therefore, *L*. *minor/gibba* and *R*. *natans* are less competitive and they are able to coexist. Phosphorus history drives growth and competition of free-floating plants.

## Supporting Information

S1 Table P and N concentrations in media of the monocultures of *Azolla*, *Lemna* and *Riccocarpus* at the start and end of the experiment and the amount of nutrients used. (DOCX)

S2 Table P and N concentrations in media of combinations of *Azolla* and *Lemna* and *Lemna* and *Ricciocarpus* at the start and at the end of the experiment and the amounts used. (DOCX)

S3 Table Basic data concerning the relative growth rate for the different species in monocultures and in mixtures. (DOCX)

S4 Table P and N concentrations in plants from monocultures at the end of the experiment. (DOCX)
