# Supplementary material for: Competition between Free-Floating Plants Is Strongly Driven by Previously Experienced Phosphorus Concentrations in the Water Column
Source: PLoS One. 2016 Sep 13;11(9):e0162780. doi: 10.1371/journal.pone.0162780 (PMC5021290; doi:10.1371/journal.pone.0162780)
Supplement: S1 Table — (DOCX) [file pone.0162780.s001.docx]

S1 Table. P and N concentrations in media of the monocultures of *Azolla*, *Lemna* and *Riccocarpus* at the start and end of the experiment and the amount of nutrients used.

|  | |  |  | Phosphate (mg P/L) | | | Nitrogen (mg N/L) | | |
| --- | --- | --- | --- | --- | --- | --- | --- | --- | --- |
| Species | P history | Present P | Pot | Start | End | Used | Start | End | Used |
| *Azolla* | high | high | 5 | 1.67 | 0.06 | 1.61 | 4.13 | 1.47 | 2.66 |
|  | high | high | 65 | 1.66 | 0.15 | 1.51 | 4.33 | 3.14 | 1.18 |
|  | low | high | 19 | 1.67 | 0.65 | 1.03 | 4.13 | 2.30 | 1.83 |
|  | low | high | 75 | 1.63 | 0.94 | 0.69 | 4.28 | 2.66 | 1.62 |
|  | high | low | 29 | 0.03 | 0.01 | 0.02 | 3.99 | 2.58 | 1.40 |
|  | high | low | 59 | 0.04 | 0.01 | 0.03 | 3.57 | 2.99 | 0.58 |
|  | low | low | 43 | 0.04 | 0.01 | 0.03 | 3.57 | 3.90 | -0.34 |
|  | low | low | 51 | 0.04 | 0.01 | 0.03 | 3.57 | 4.09 | -0.52 |
| *Lemna* | high | high | 3 | 1.67 | 0.33 | 1.34 | 4.13 | 0.13 | 4.00 |
|  | high | high | 63 | 1.66 | 0.30 | 1.36 | 4.33 | 0.21 | 4.12 |
|  | high | high | 77 | 1.63 | 0.42 | 1.22 | 4.28 | 0.34 | 3.94 |
|  | low | high | 21 | 1.67 | 0.34 | 1.33 | 4.13 | 0.06 | 4.07 |
|  | high | low | 35 | 0.03 | 0.01 | 0.02 | 3.99 | 0.43 | 3.56 |
|  | high | low | 37 | 0.04 | 0.01 | 0.03 | 3.57 | 0.48 | 3.08 |
|  | low | low | 33 | 0.03 | 0.01 | 0.03 | 3.99 | 2.90 | 1.09 |
| *Ricciocarpus* | high | high | 5 | 1.65 | 0.85 | 0.80 | 4.13 | 0.47 | 3.67 |
|  | low | high | 19 | 1.65 | 0.83 | 0.82 | 4.13 | 1.69 | 2.44 |
|  | high | low | 27 | 0.04 | 0.00 | 0.03 | 4.23 | 1.29 | 2.93 |
|  | low | low | 39 | 0.04 | 0.00 | 0.03 | 4.23 | 3.58 | 0.65 |
