# Supplementary material for: Competition between Free-Floating Plants Is Strongly Driven by Previously Experienced Phosphorus Concentrations in the Water Column
Source: PLoS One. 2016 Sep 13;11(9):e0162780. doi: 10.1371/journal.pone.0162780 (PMC5021290; doi:10.1371/journal.pone.0162780)
Supplement: S2 Table — (DOCX) [file pone.0162780.s002.docx]

S2 Table. P and N concentrations in media of combinations of *Azolla* and *Lemna* and *Lemna* and *Ricciocarpus* at the start and at the end of the experiment and the amounts used.

|  | |  |  | Phosphate (mg P/L) | | | Nitrogen (mg N/L) | | |
| --- | --- | --- | --- | --- | --- | --- | --- | --- | --- |
| Combination | P history | Present P | Pot | Start | End | Used | Start | End | Used |
| *Azolla Lemna* | high high | high | 61 | 1.66 | 0.03 | 1.63 | 4.33 | 1.32 | 3.01 |
| *Azolla Lemna* | high low | high | 11 | 1.67 | 0.46 | 1.21 | 4.13 | 1.06 | 3.07 |
| *Azolla Lemna* | high low | high | 71 | 1.63 | 0.30 | 1.33 | 4.28 | 0.49 | 3.79 |
| *Azolla Lemna* | low high | high | 13 | 1.67 | 0.08 | 1.59 | 4.13 | 0.15 | 3.98 |
| *Azolla Lemna* | low low | high | 23 | 1.67 | 0.29 | 1.38 | 4.13 | 1.03 | 3.1 |
| *Azolla Lemna* | low high | low | 25 | 0.03 | 0.01 | 0.02 | 3.99 | 2.34 | 1.65 |
| *Azolla Lemna* | high high | low | 31 | 0.03 | 0.01 | 0.02 | 3.99 | 1.07 | 2.92 |
| *Azolla Lemna* | high high | low | 57 | 0.04 | 0.01 | 0.03 | 3.57 | 1.83 | 1.74 |
| *Azolla Lemna* | high low | low | 39 | 0.04 | 0.01 | 0.03 | 3.57 | 1.97 | 1.59 |
| *Azolla Lemna* | high low | low | 53 | 0.04 | 0.01 | 0.03 | 3.57 | 1.98 | 1.59 |
| *Azolla Lemna* | low low | low | 45 | 0.04 | 0.08 | 0.03 | 3.57 | 3.71 | -0.14 |
| *Lemna Ricciocarpus* | high high | high | 1 | 1.65 | 0.52 | 1.14 | 4.13 | 0.18 | 3.96 |
| *Lemna Ricciocarpus* | high low | high | 11 | 1.65 | 0.58 | 1.07 | 4.13 | 0.48 | 3.65 |
| *Lemna Ricciocarpus* | low high | high | 13 | 1.65 | 0.49 | 1.16 | 4.13 | 0.15 | 3.98 |
| *Lemna Ricciocarpus* | low low | high | 23 | 1.65 | 0.66 | 0.99 | 4.13 | 0.89 | 3.24 |
| *Lemna Ricciocarpus* | high high | low | 29 | 0.04 | 0.01 | 0.03 | 4.23 | 0.84 | 3.39 |
| *Lemna Ricciocarpus* | high low | low | 37 | 0.04 | 0.01 | 0.03 | 4.23 | 1.61 | 2.61 |
| *Lemna Ricciocarpus* | low high | low | 25 | 0.04 | 0.01 | 0.03 | 4.23 | 1.45 | 2.78 |
