# Supplementary material for: Competition between Free-Floating Plants Is Strongly Driven by Previously Experienced Phosphorus Concentrations in the Water Column
Source: PLoS One. 2016 Sep 13;11(9):e0162780. doi: 10.1371/journal.pone.0162780 (PMC5021290; doi:10.1371/journal.pone.0162780)
Supplement: S3 Table — (DOCX) [file pone.0162780.s003.docx]

S3 Table. Basic data concerning the relative growth rate for the different species in monocultures and in mixtures.

*Monocultures*

| Plant species | P history | Present P | Series | Exp Unit | RGR (g/g/d) |
| --- | --- | --- | --- | --- | --- |
| Azolla | High | High | 1 | 5 | 0.0762 |
|  |  |  |  | 6 | 0.0577 |
|  |  |  | 2 | 65 | 0.0729 |
|  |  |  |  | 66 | 0.0609 |
|  | High | Low | 1 | 29 | 0.0815 |
|  |  |  |  | 30 | 0.0666 |
|  |  |  | 2 | 59 | 0.0861 |
|  |  |  |  | 60 | 0.0676 |
|  | Low | High | 1 | 19 | 0.0282 |
|  |  |  |  | 20 | 0.0273 |
|  |  |  | 2 | 75 | 0.0180 |
|  |  |  |  | 76 | 0.0099 |
|  | Low | Low | 1 | 43 | 0.0078 |
|  |  |  |  | 44 | 0.0142 |
|  |  |  | 2 | 51 | 0.0134 |
|  |  |  |  | 52 | 0.0107 |
| Lemna | High | High | 1 | 3 | 0.0546 |
|  |  |  |  | 4 | 0.0629 |
|  |  |  | 2 | 63 | 0.0676 |
|  |  |  |  | 64 | 0.0720 |
|  |  |  | 3 | 77 | 0.0592 |
|  |  |  |  | 78 | 0.0633 |
|  | High | Low | 1 | 35 | 0.0604 |
|  |  |  |  | 36 | 0.0631 |
|  |  |  | 2 | 37 | 0.0662 |
|  |  |  |  | 38 | 0.0703 |
|  | Low | High | 1 | 21 | 0.0262 |
|  |  |  |  | 22 | 0.0283 |
|  | Low | Low | 1 | 33 | 0.0067 |
|  |  |  |  | 34 | 0.0065 |
| Lemna* | High | High | 4 | 3 | 0.1019 |
|  |  |  |  | 4 | 0.0996 |
|  | High | Low | 3 | 35 | 0.1048 |
|  |  |  |  | 36 | 0.1098 |
| Ricciocarpus | High | High | 1 | 5 | 0.0704 |
|  |  |  |  | 6 | 0.0680 |
|  | High | Low | 1 | 27 | 0.0906 |
|  |  |  |  | 28 | 0.0991 |
|  | Low | High | 1 | 19 | 0.1279 |
|  |  |  |  | 20 | 0.1331 |
|  | Low | Low | 1 | 39 | 0.1123 |
|  |  |  |  | 40 | 0.1123 |
| Lemna*: monocultures used in experiment with Ricciocarpus | | | | |  |

*Mixtures*

|  | Plant species | P history | Present P | Series | Exp pot^#^ | RGR (g/g/d) |
| --- | --- | --- | --- | --- | --- | --- |
|  | Azolla | Azolla High & Lemna High | High | 1 | 1B | 0.0902 |
|  |  |  |  |  | 2B | 0.0941 |
|  |  |  |  | 2 | 61B | 0.1046 |
|  |  |  |  |  | 62B | 0.1058 |
|  |  | Azolla High & Lemna Low | High | 1 | 13B | 0.1039 |
|  |  |  |  |  | 14B | 0.1032 |
|  |  | Azolla Low & Lemna High | High | 1 | 11B | 0.0328 |
|  |  |  |  |  | 12B | 0.0308 |
|  |  |  |  | 2 | 71B | 0.0299 |
|  |  |  |  |  | 72B | 0.0397 |
|  |  | Azolla Low & Lemna Low | High | 1 | 23B | 0.0487 |
|  |  |  |  |  | 24B | 0.0383 |

*Mixtures continued*

|  | Plant species | P history | Present P | Series | Exp pot^#^ | RGR (g/g/d) |
| --- | --- | --- | --- | --- | --- | --- |
|  | Azolla | Azolla High & Lemna High | Low | 1 | 31B | 0.0956 |
|  |  |  |  |  | 32B | 0.0984 |
|  |  |  |  | 2 | 57B | 0.1138 |
|  |  |  |  |  | 58B | 0.0943 |
|  |  | Azolla High & Lemna Low | Low | 1 | 25B | 0.0988 |
|  |  |  |  |  | 26B | 0.1005 |
|  |  | Azolla Low & Lemna High | Low | 1 | 39B | 0.0087 |
|  |  |  |  |  | 40B | 0.0132 |
|  |  |  |  | 2 | 53B | 0.0107 |
|  |  |  |  |  | 54B | 0.0037 |
|  |  | Azolla Low & Lemna Low | Low | 1 | 45B | 0.0040 |
|  |  |  |  |  | 46B | 0.0055 |
|  | Lemna | Lemna High & Azolla High | High | 1 | 1A | 0.0444 |
|  |  |  |  |  | 2A | 0.0459 |
|  |  |  |  | 2 | 61A | 0.0570 |
|  |  |  |  |  | 62A | 0.0572 |
|  |  | Lemna High & Azolla Low | High | 1 | 11A | 0.0665 |
|  |  |  |  |  | 12A | 0.0685 |
|  |  |  |  | 2 | 71A | 0.0685 |
|  |  |  |  |  | 72A | 0.0603 |
|  |  | Lemna Low & Azolla High | High | 1 | 13A | 0.0135 |
|  |  |  |  |  | 14A | 0.0260 |
|  |  | Lemna Low & Azolla Low | High | 1 | 23A | 0.0269 |
|  |  |  |  |  | 24A | 0.0262 |
|  |  | Lemna High & Azolla High | Low | 1 | 31A | 0.0537 |
|  |  |  |  |  | 32A | 0.0518 |
|  |  |  |  | 2 | 57A | 0.0532 |
|  |  |  |  |  | 58A | 0.0531 |
|  |  | Lemna High & Azolla Low | Low | 1 | 39A | 0.0681 |
|  |  |  |  |  | 40A | 0.0670 |
|  |  |  |  | 2 | 53A | 0.0714 |
|  |  |  |  |  | 54A | 0.0724 |
|  |  | Lemna Low & Azolla High | Low | 1 | 25A | -0.0015 |
|  |  |  |  |  | 26A | -0.0040 |
|  |  | Lemna Low & Azolla Low | Low | 1 | 45A | 0.0011 |
|  |  |  |  |  | 46A | 0.0020 |
|  |  | Lemna High & Ricciocarpus High | High | 1 | 1A | 0.0741 |
|  |  |  |  |  | 2A | 0.0727 |
|  |  | Lemna High & Ricciocarpus Low | High | 1 | 11A | 0.0770 |
|  |  |  |  |  | 12A | 0.0780 |
|  |  | Lemna Low & Ricciocarpus High | High | 1 | 13A | 0.0293 |
|  |  | Lemna Low & Ricciocarpus Low | High | 1 | 23A | 0.0234 |
|  |  | Lemna High & Ricciocarpus High | Low | 1 | 29A | 0.0670 |
|  |  | Lemna High & Ricciocarpus Low | Low | 1 | 37A | 0.0714 |
|  |  | Lemna Low & Ricciocarpus High | Low | 1 | 25A | -0.0026 |
|  | Ricciocarpus | Ricciocarpus Hhigh & Lemna High | High | 1 | 1B | 0.0150 |
|  |  |  |  |  | 2B | 0.0368 |
|  |  | Ricciocarpus High & Lemna Low | High | 1 | 13B | 0.0522 |
|  |  | Ricciocarpus Low & Lemna High | High | 1 | 11B | -0.0468 |
|  |  |  |  |  | 12B | -0.0776 |
|  |  | Ricciocarpus Low & Lemna Low | High | 1 | 23B | -0.0520 |
|  |  | Ricciocarpus High & Lemna High | Low | 1 | 29B | 0.0065 |
|  |  |  |  |  | 30B | 0.0054 |
|  |  | Ricciocarpus High & Lemna Low | Low | 1 | 25B | 0.0368 |
|  |  | Ricciocarpus Low & Lemna High | Low | 1 | 37B | -0.0251 |
|  |  |  |  |  | 38B | -0.0208 |
|  | ^#^: pots with the same number and a different letter belong to the same treatment | | | | |  |
