# Supplementary material for: Competition between Free-Floating Plants Is Strongly Driven by Previously Experienced Phosphorus Concentrations in the Water Column
Source: PLoS One. 2016 Sep 13;11(9):e0162780. doi: 10.1371/journal.pone.0162780 (PMC5021290; doi:10.1371/journal.pone.0162780)
Supplement: S4 Table — (DOCX) [file pone.0162780.s004.docx]

S4 Table. P and N concentrations in plants from each monoculture at the end of the experiment.

|  |  |  | P mg/g DW | | | | N mg/g DW | | | |
| --- | --- | --- | --- | --- | --- | --- | --- | --- | --- | --- |
| Species | P history | P present | 1 | 2 | 3 | 4 | 1 | 2 | 3 | 4 |
| *Azolla* | high | high | 8.7 | 7.4 | 9.0 | 8.8 | 51.5 | 49.4 | 49.7 | 50.0 |
|  | high | low | 2.6 | 2.7 | 2.2 | 2.9 | 46.6 | 47.7 | 45.2 | 47.3 |
|  | low | high | 4.1 | 4.2 | 3.3 | 3.7 | 38.6 | 42.1 | 37.5 | 37.2 |
|  | low | low | 0.3 | 0.3 | 0.3 | 0.3 | 33.5 | 36.0 | 31.7 | 35.0 |
| *Lemna* | high | high | 7.1 | 7.6 | 7.4 | 7.8 | 21.9 | 23.2 | 26.5 | 27.8 |
|  | high | low | 2.5 | 2.7 | 2.6 | 2.6 | 23.9 | 23.8 | 29.4 | 28.1 |
|  | low | high | 4.2 | 4.5 |  |  | 21.8 | 24.3 |  |  |
|  | low | low | 0.1 |  |  |  | 23.4 |  |  |  |
| *Riccocarpus* | high | high | 7.1 | 7.6 |  |  | 40.4 | 42.2 |  |  |
|  | high | low | 2.1 | 2.1 |  |  | 38.5 | 38.3 |  |  |
|  | low | high | 5.2 | 5.2 |  |  | 58.4 | 56.7 |  |  |
|  | low | low | 0.4 | 0.4 |  |  | 45.3 | 43.0 |  |  |
